# Histological and Morphological Evaluation of Exfoliated Silver Diamine Fluoride-Treated Carious Primary Teeth

**DOI:** 10.7759/cureus.83554

**Published:** 2025-05-06

**Authors:** Mridula Goswami, Riya Marie Johnson, Babita Jangra

**Affiliations:** 1 Pediatric and Preventive Dentistry, Maulana Azad Institute of Dental Sciences, New Delhi, IND

**Keywords:** caries, histology, remineralization, scanning electron microscope, silver diamine fluoride

## Abstract

Silver diamine fluoride (SDF) is a minimally invasive treatment modality that has gained attention in recent years owing to its remineralizing capability. However, there is limited research on its effect at a microscopic level. This case series presents three cases of exfoliated primary teeth where histologic and morphological analysis was performed after a single application of SDF on carious dentin. This paper provides valuable insights into the long-term and sustained effectiveness of SDF, making it a cost-effective treatment for young, uncooperative children including those with special health care needs. In addition, it also highlights that periodic re-application of SDF can enhance the overall caries-arresting potential. However, despite the clinical success, the inclusion of silver particles deep within the dentinal tubules results in pronounced discoloration, which is a significant drawback of SDF. Future research has to be directed toward innovative techniques to overcome discoloration while maintaining its efficacy.

## Introduction

Dental caries is the most prevalent oral disease worldwide, affecting approximately 3.5 billion people, with a global burden of 514 million children affected by caries in their primary teeth [1]. The traditional caries management strategies are difficult in young, uncooperative patients and children with special health care needs owing to factors such as fear and anxiety, short attention span, limited cooperation, and behavioral challenges. Silver diamine fluoride (SDF) is a minimally invasive, topical antimicrobial and remineralizing agent that was approved by the Food and Drug Administration (FDA) in 2014 [2]. It is a safe, quick, effective, cost-effective, and child-friendly preventive treatment approach that halts the progression of active carious lesions [3].

SDF is distinguished by its exceptionally high fluoride (44,800 ppm) and silver (2,55,000 ppm) concentration that exerts a synergistic effect and its unique ability to arrest and potentially reverse active carious lesions [4]. The mechanism of action of SDF can be attributed to its antibacterial properties, which target bacterial cellular structures and the metabolic processes of cariogenic microflora; its remineralizing effect on the inorganic content of the tooth through the formation of fluorapatite; and its inhibitory effect on the degradation of the organic matrix by suppressing matrix metalloproteinases [5]. However, black discoloration remains one of the major drawbacks, raising aesthetic concerns and thereby limiting its use [6,7].

To assess the long-term outcomes and success of SDF at the microscopic and structural levels, it is essential to conduct histological and morphological evaluations. These assessments help determine the depth of remineralization, ion distribution, and integrity of tooth structure following SDF treatment. This unique case series presents a histological and morphological evaluation of carious primary teeth that were treated with SDF and later exfoliated after a prolonged period. It discusses three cases involving four exfoliated carious primary teeth, two analyzed through histological evaluation and two examined using scanning electron microscopy (SEM). The aim of this case series is to assess the long-term effects of SDF on tooth structure, specifically focusing on mineralization, dentin integrity, and tertiary dentin formation. By combining histological and SEM analyses, this paper provides valuable insights into the sustained impact of SDF on caries arrest and remineralization, contributing to a deeper understanding of its effectiveness as a non-invasive treatment in pediatric dentistry.

## Case presentation

Case 1

**Figure 1:**
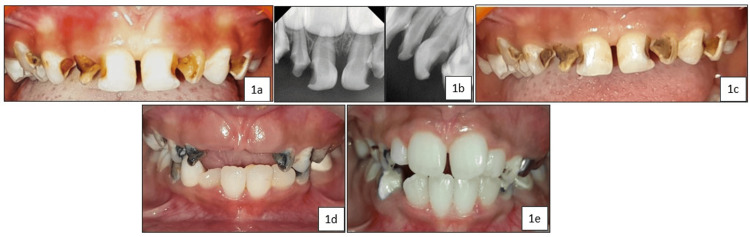
(a) Pre-operative image showing caries with an International Caries Detection and Assessment System II (ICDAS II) score of 5 in teeth 51, 52, 53, 54, 61, 62, 63, and 64; (b) Pre-operative radiograph showing deep dentinal caries in teeth 51, 52, 53, 54, 61, 62, 63, and 64; (c) Immediate post-operative image following silver diamine fluoride application; (d) 12-month follow-up showing exfoliated teeth 51 and 61; (e) 27-month follow-up showing erupting teeth 11, 12, 21, and 22.

A five-year-old female patient presented to our department with severe early childhood caries persisting for the past six months. The child was otherwise healthy and showed no associated symptoms. Clinical and radiographic examinations revealed an International Caries Detection and Assessment System (ICDAS) II score of 5 for teeth 51, 52, 53, 54, 61, 62, 63, and 64 (Figure 1a and Figure 1b). The child was classified as Frankl’s Behaviour Rating Scale 2 (negative) and was uncooperative during the examination. Therefore, with parental consent, a minimally invasive treatment involving a single application of SDF (e-SDF Kids, e-Dental LLP, Mumbai, India) was performed on all carious teeth (Figure 1c). During follow-up visits, the child remained asymptomatic. Over time, the primary anterior teeth exfoliated, and tooth 52 was retrieved by the parent immediately post-exfoliation, 24 months after SDF treatment (Figure 1d and Figure 1e). The tooth was immersed in 10% buffered formalin and decalcified using 10% nitric acid. After decalcification, the specimen was thoroughly rinsed, dehydrated through graded ethanol solutions, cleared with xylene, and embedded in molten paraffin wax. Tooth sections (3-5 µm thick) were then prepared, stained with Hematoxylin and Eosin, and examined histologically under light microscopy.

Histologic Examination

**Figure 2:**
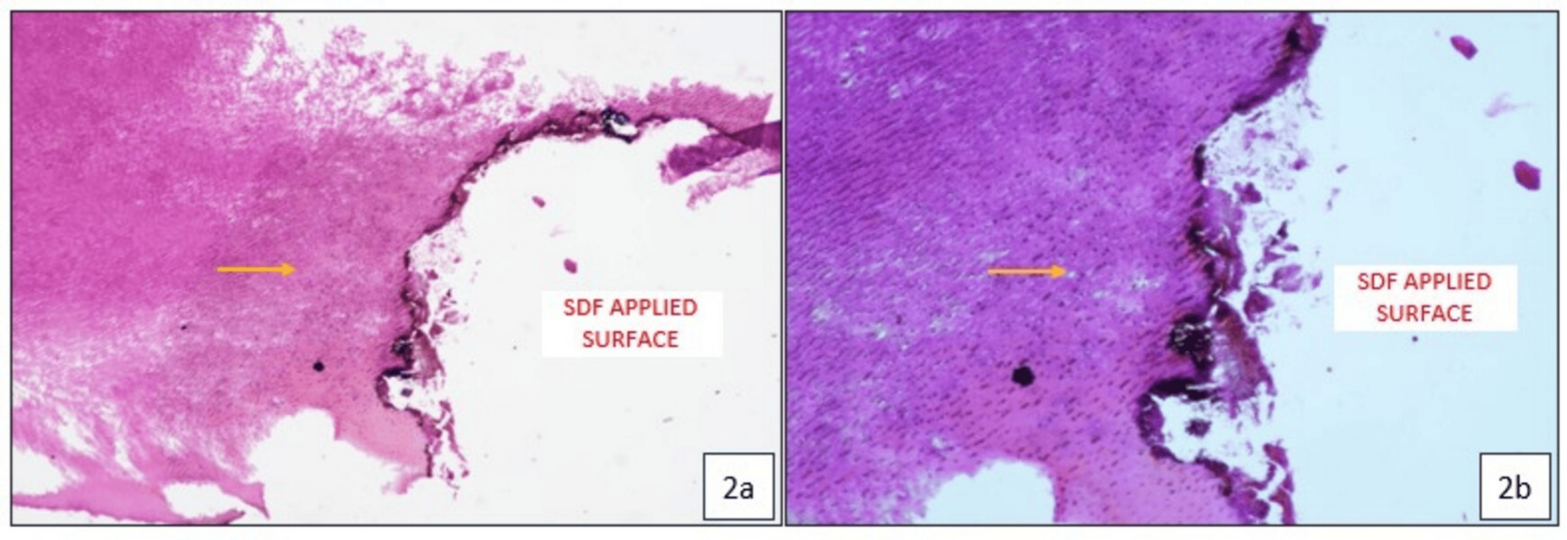
Histopathological images of exfoliated tooth 52 showing the carious surface treated with silver diamine fluoride and dense silver particle penetration within the dentinal tubules (yellow arrow), resulting in effective tubule occlusion: (a) 10× magnification; (b) 20× magnification.

Histological examination shows a dark-stained carious tooth surface at the site of SDF application, with normal dentin structure observed adjacent to the carious area. Dense silver particle inclusions are seen penetrating deeply throughout the dentinal tubules, with no evidence of bacterial presence (Figure 2a and Figure 2b).

Case 2

**Figure 3:**
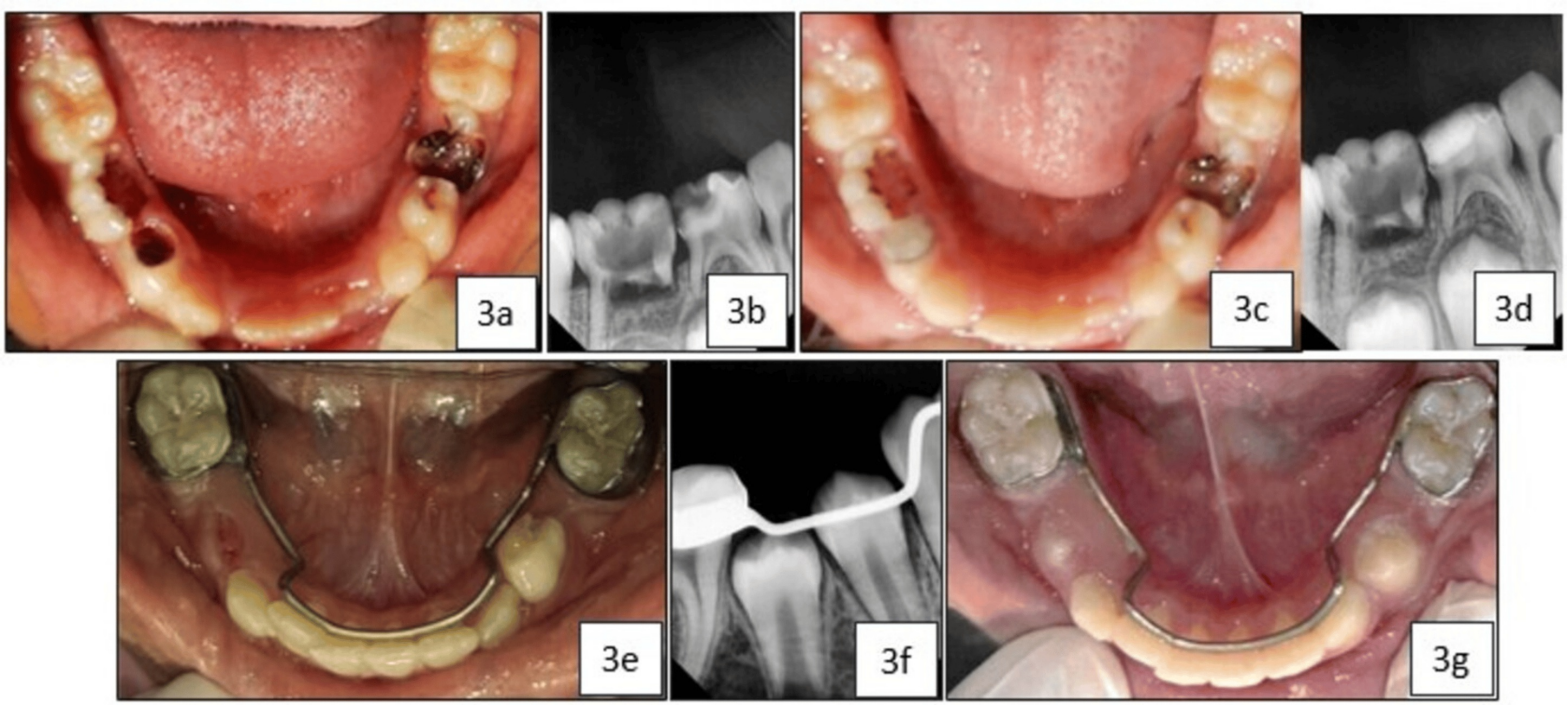
a) Pre-operative image showing International Caries Detection and Assessment System (ICDAS) II score 3 for tooth 74, score 5 for tooth 84, and score 6 for teeth 75 and 85; (b) Pre-operative radiograph showing teeth 84 and 85; (c) Immediate post-operative view showing silver diamine fluoride (SDF) and glass ionomer cement (GIC) applied using the silver-modified atraumatic restorative treatment (SMART) technique on tooth 84; (d) Post-operative radiograph showing tooth 84; (e) 29-month follow-up image showing exfoliated tooth 84; (f) 29-month follow-up radiograph showing erupting teeth 44 and 45; (g) 31-month follow-up image showing erupting teeth 34 and 44.

A seven-year-old female patient presented to our department with the chief complaint of multiple decayed teeth. The patient was healthy with no relevant medical history. Clinical and radiographic examinations revealed deep dentinal caries in the primary right mandibular first molar (tooth 84), classified as ICDAS II score 5 (Figure 3a and Figure 3b). Tooth 74 was classified as ICDAS II score 3, while teeth 75 and 85 were classified as ICDAS II score 6 (Figure 3a). With parental consent, a minimally invasive treatment using the silver-modified atraumatic restorative treatment (SMART) technique was performed on tooth 84. This involved the application of SDF (e-SDF Kids, e-Dental LLP, Mumbai, India) followed by restoration with light-cured glass ionomer cement (GIC; GC Corporation, Tokyo, Japan) (Figure 3c and Figure 3d). Tooth 74 was restored with light-cured GIC, while teeth 75 and 85 were extracted under local anesthesia, followed by the placement of a lingual arch space maintainer. Tooth 84 remained clinically and radiographically asymptomatic during follow-up visits and exfoliated naturally after 29 months (Figure 3e, Figure 3f, and Figure 3g). The exfoliated tooth was immediately retrieved from the parent, fixed in 10% buffered formalin, and sectioned for histological examination using hematoxylin and eosin staining under light microscopy.

Histologic Examination

**Figure 4:**
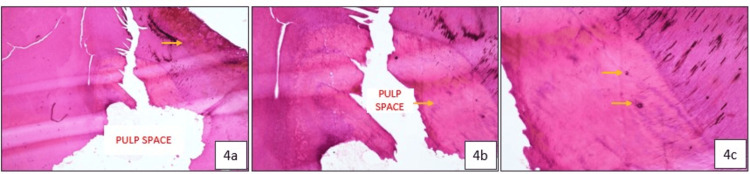
Histopathological images of exfoliated tooth 84 showing the carious surface treated with Silver Diamine Fluoride (SDF) and dense silver particle penetration deep within the tertiary dentin: (a) 4× magnification (yellow arrow indicating SDF penetration within the dentinal tubules); (b) 10× magnification (yellow arrow indicating disorganized and irregular tertiary dentin formation adjacent to the pulp space); (c) 20× magnification (yellow arrow indicating silver particle inclusion within the tertiary dentin).

The histological examination revealed a dark-stained carious tooth surface at the site of SDF application, with normal dentin structure observed adjacent to the carious surface (Figure 4a). An irregular and disorganized layer of tertiary dentin formation was also observed adjacent to the pulp space (Figure 4b). Dense silver particle inclusions were seen penetrating deeply through the dentinal tubules (Figure 4a), extending as far as the tertiary dentin, with no evidence of bacterial presence (Figure 4c).

Case 3

**Figure 5:**
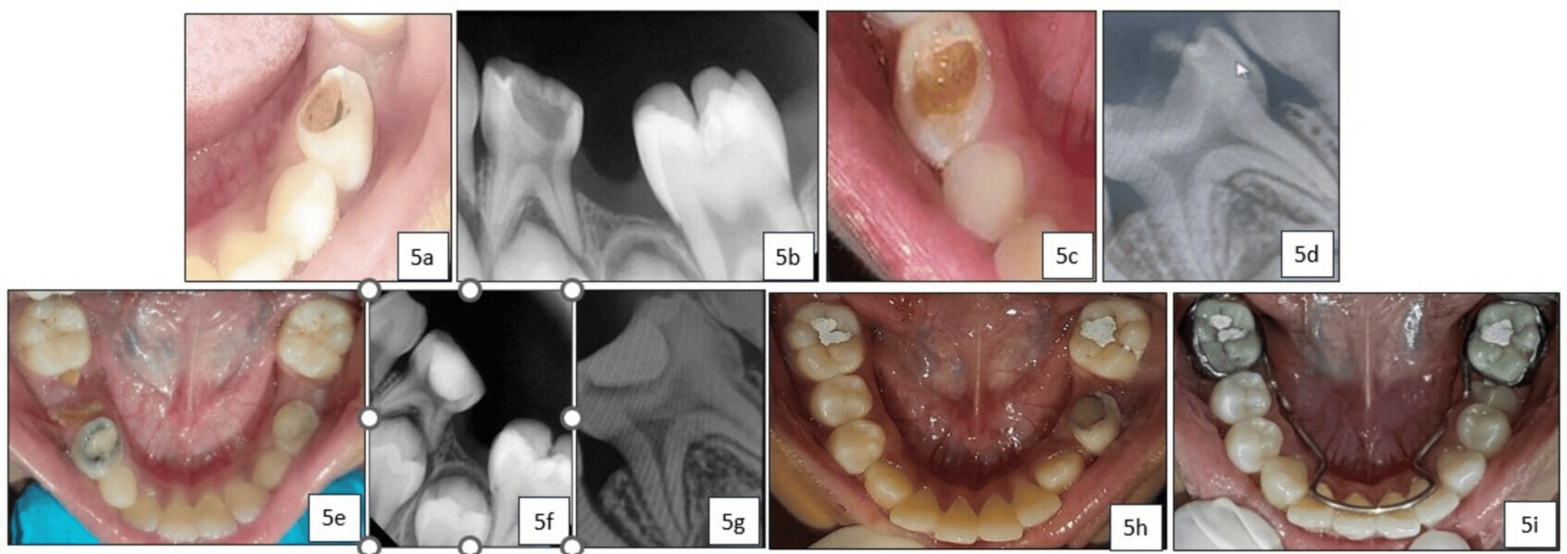
(a) Pre-operative image showing International Caries Detection and Assessment System (ICDAS) II score 5 in relation to 74; (b) Pre-operative radiograph in relation to 74; (c) Pre-operative image showing ICDAS II score 5 in relation to 84; (d) Pre-operative radiograph in relation to 84; (e) Immediate post-operative clinical view showing silver diamine fluoride + glass ionomer cement (silver-modified atraumatic restorative treatment technique) applied in relation to 74 and 84; (f) Post-operative radiograph of 74; (g) Post-operative radiograph of 84; (h) 12-month follow-up showing exfoliated 84 with erupting 44 and 45; (i) 24-month follow-up showing lingual arch space maintainer with erupting 34 and 35.

An eight-year-old female patient presented to our department with a chief complaint of decayed lower right and left posterior teeth. Upon clinical and radiographic examination, deep dentinal caries were observed in the primary left (74) and right (84) mandibular first molars, classified as ICDAS II score 5 (Figure 5a, Figure 5b, Figure 5c, and Figure 5d). Following parental consent, treatment was performed using the SMART technique with SDF (e-SDF Kids-e-Dental, Llp, Mumbai, India) and light-cure GIC (GC Corporation, Tokyo, Japan) (Figure 5e, Figure 5f, and Figure 5g). The teeth were asymptomatic during follow-up visits, and after 12 months, both teeth underwent normal exfoliation (Figure 5h and Figure 5i) and were retrieved for morphological analysis using SEM.

Sample Preparation for SEM Analysis

Each tooth sample was sectioned through the carious lesions into two halves, each approximately 4-5 mm thick, using a carborundum disc. During preparation, the GIC in the SMART technique samples was dislodged due to mechanical stress. The samples were then analyzed using an SEM for qualitative assessment (JSM 6610LV Scanning Electron Microscope, USIC, Delhi).

SEM Analysis

**Figure 6:**
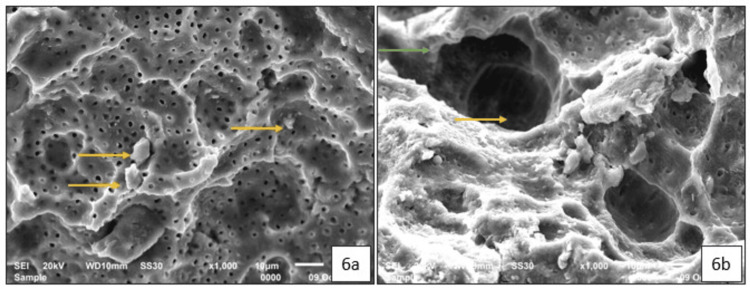
Scanning electron micrographs of 74 after 12 months of exfoliation: (a) 1000X magnification of the silver diamine fluoride-treated carious dentin, showing multiple areas of hypermineralized inter-tubular dentin (yellow arrow); (b) 1000X magnification showing hypermineralized peritubular dentin (green arrow) and partial occlusion of the dentinal tubules (yellow arrow).

Tooth 74: The SEM images of the specimen revealed the presence of multiple hypermineralized globular micro-aggregates within the peritubular dentin (Figure 6a) and intra-tubular dentin (Figure 6b). The SDF-treated carious dentin surface appears relatively smooth, with evidence of dentinal tubule occlusion (Figure 6b).

**Figure 7:**
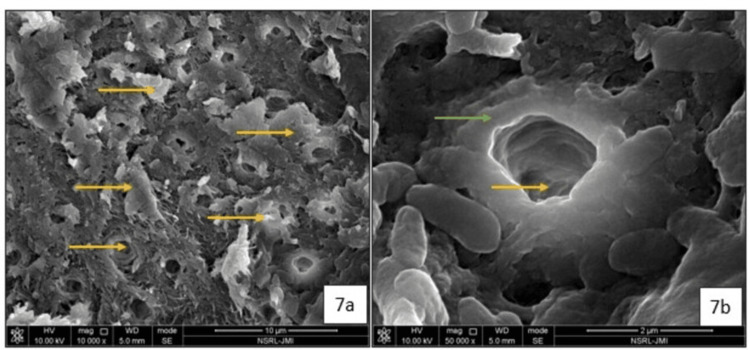
Scanning Electron Micrographs of 84 after 12 months of exfoliation (a) 10,000X magnification of the Silver Diamine Fluoride treated carious dentin showing multiple areas of hypermineralised intertubular dentin (yellow arrow); (b) 50,000X magnification showing hypermineralised peritubular dentin (green arrow) and partial occlusion of the dentinal tubules (yellow arrow).

Tooth 84: The SEM images of the specimen revealed multiple hypermineralized micro-aggregates within the peritubular dentin (Figure 7a) and intra-tubular dentin (Figure 7b), with partial occlusion of the dentinal tubules (Figure 7b).

Treatment procedure

Prior to the commencement of treatment, the procedure was explained to the parents and their child, including the application of SDF, potential adverse effects, and possible discoloration. Written informed consent from the parents and assent from the child were obtained. Gross debris was removed from the carious lesion to ensure optimal penetration of SDF into the carious tooth structure. Petroleum jelly was applied to the lips and gingiva to minimize the risk of temporary staining. The area adjacent to the treated tooth was isolated using cotton rolls, and the carious lesion was dried with a gentle stream of compressed air. One drop of SDF solution (e-SDF, Kids-e-Dental, LLP, Mumbai, India) was placed in a disposable plastic dappen dish. A microbrush was used to apply the SDF, ensuring excess liquid was removed by gently dabbing the brush on the side of the dappen dish before application. The SDF was applied continuously for one minute, with agitation using the microbrush, and isolation was maintained for a total of three minutes. Excess SDF was removed using gauze or cotton rolls, and the tooth was examined for a noticeable color change in the carious lesion. In the SMART technique, the tooth was restored with light-cure GIC (GC Corporation, Tokyo, Japan) during the same visit. The child and parents were then provided with instructions on oral hygiene, diet modifications, and the importance of regular follow-up visits.

## Discussion

This case series represents the first reported investigation providing microscopic insights into the long-term effects of SDF on exfoliated primary teeth. Despite the considerable time lapse between treatment and evaluation, the SDF-treated teeth exhibited evidence of caries arrest and remineralization. In this case series, two primary teeth were evaluated for histological examination approximately two years after a single application of SDF.

Histological analysis revealed the presence of dense silver particle precipitation within the dentinal tubules, resulting in effective occlusion. There were signs of continued preservation of the integrity of the dentin, with intact peritubular and inter-tubular dentin and no bacterial infiltration. The silver component in SDF causes direct inhibition of bacterial colonization and mechanical sealing of carious and sound dentinal tubules, thereby preventing carious progression. Further, the placement of GIC provides additional sealing against microbial ingress [8]. Newly formed, disorganized, and irregular tertiary dentin was observed adjacent to the pulp space in one of the cases, suggesting an active odontoblastic response to the caries. This indicates the functional activity of odontoblasts in an attempt to protect the pulp and limit further progression of the caries.

Silver particles were also seen penetrating deep, with silver inclusions detected within the tertiary dentin in one of our cases, highlighting the deep antimicrobial effects. The extent of silver ion penetration has been reported variably in different studies. Chu et al. (2008) reported silver penetration depths of up to 25 to 200 µm, whereas Bimstein et al. (2018), in a case report, reported an SDF penetration depth of up to 1 mm [9,10]. Li et al. (2019) reported an average penetration depth of 744.65 ± 448.69 µm within the dentinal tubules of the carious lesion. The increased depth of silver penetration can be attributed to factors such as the proximity of the cavity to the pulp, the young age of the patient, and the increased diameter of the dentinal tubules in this area [11]. Sayed et al. (2019) concluded that silver penetration depth increased with the depth of demineralization and time [12].

One of the disadvantages of SDF is the black discoloration resulting from the formation of silver phosphate by the reaction of silver ions with hydroxyapatite. Silver phosphate, being unstable, quickly reduces back into silver ions, which then interact with the exposed collagen in the demineralized dentin, leading to the formation of metallic silver and resulting in discoloration [13]. The greater the amount and depth of collagen exposed due to demineralization, the greater the SDF penetration and more silver ions are reduced into metallic silver in a shorter period [12]. Hence, eliminating the discoloration without compromising its therapeutic effects remains a significant challenge and highlights the need for future research and advanced innovation.

The histological appearance of dentinal tubule occlusion can be further supported with SEM analysis. In one of our cases, SEM analysis was conducted on two SDF-treated primary teeth that exfoliated after a period of 12 months. Scanning Electron Micrographs demonstrated the partial occlusion of dentinal tubules by silver particles and smooth remineralized deposits, validating the histological findings and extent of occlusion. Sayed et al. (2019) observed that in the demineralized dentin groups, multiple crystal formations were found beneath the surface, with their number gradually decreasing toward the pulpal direction [12]. Previous studies have speculated that multiple discrete crystal formations after SDF application are likely silver crystals, as analyzed by energy-dispersive X-ray spectroscopy [14]. The partially obliterated dentinal tubules can be correlated to the improvement of dentin hardness [15].

## Conclusions

This case series presents a comprehensive histological and morphological evaluation of exfoliated carious primary teeth treated with SDF. The findings demonstrate the long-term effectiveness, with evidence of sustained dentinal tubule occlusion, depth of penetration, and antimicrobial properties. However, the management of discoloration remains a significant challenge, highlighting the need for future research to develop new strategies to mitigate this cosmetic concern while maintaining its efficacy. The findings of this paper highlight the remarkable sustained efficacy of SDF as a non-invasive caries management strategy, with the potential to offer long-term benefits against further tooth demineralization. The ability of even a single application of SDF to provide persistent effects makes it a highly effective treatment option for managing carious lesions in young, uncooperative children and children with special health care needs, where traditional restorative procedures may be difficult or less feasible. Further, periodic reapplication of SDF can provide cumulative benefits and prolong the effects.
